# Area Dose–Response and Radiation Origin of Childhood Thyroid Cancer in Fukushima Based on Thyroid Dose in UNSCEAR 2020/2021: High ^131^I Exposure Comparable to Chernobyl

**DOI:** 10.3390/cancers15184583

**Published:** 2023-09-15

**Authors:** Toshiko Kato, Kosaku Yamada, Tadashi Hongyo

**Affiliations:** 1 Independent Researcher, Nara 630-8242, Japan; 2 Independent Researcher, Kyoto 611-0001, Japan; kosakuyamada@yahoo.co.jp; 3 Department of Radiation Biology, Health Sciences, Graduate School of Medicine, Osaka University, Osaka 565-0871, Japan; hongyo@sahs.med.osaka-u.ac.jp

**Keywords:** childhood thyroid cancer, ^131^I exposure, area dose–response, Fukushima, Chernobyl, excess absolute risk, radiation-induced PTC, overdiagnosis, UNSCEAR 2020/2021

## Abstract

**Simple Summary:**

After the Fukushima nuclear power plant accident in Japan on 11 March 2011, thyroid ultrasound examinations (TUEs) were conducted as part of the Fukushima health management survey (FHMS) by the Fukushima Medical University (FMU) on all residents aged ≤18 years at the time of the accident. The results showed a dozens-fold increase in thyroid cancer detection (97% papillary thyroid cancer, PTC) compared with the expected incidence from the cancer registry. More than 350 thyroid cancer patients endured poor health, being unable to disclose that they had PTC surgery due to the official stance that the nuclear accident caused no health effects. Without any international recognition of at least one adverse health effect of radiation exposure, the situation experienced by patients in Fukushima might be experienced by people exposed in the next nuclear accident. Young patients should be able to undergo appropriate operations and receive the most efficient treatments with sympathy and support from both government and society.

**Abstract:**

The FMU and the United Nations Scientific Committee on the Effects of Atomic Radiation (UNSCEAR) concluded that the high incidence of thyroid cancer after the Fukushima nuclear accident was not the result of radiation exposure, but rather might have been overdiagnosis based on the low thyroid dose estimated in the UNSCEAR 2020/2021 report. In this study, the origin of increased PTC in Fukushima was examined based on the thyroid dose estimated by UNSCEAR. The dose–response relationship of the incidence rate per person-years (PY) was analyzed for four areas in Fukushima prefecture via regression analysis. The linear response of the annual incidence rates to thyroid dose in the first six years showed that the dominant origin of childhood thyroid cancer was radiation exposure. Excess absolute risk (EAR) proportionally increased with thyroid dose, with an EAR/10^4^ PY Gy of 143 (95%CI: 122, 165) in the second TUE (*p* < 0.001), which is approximately 50–100 times higher than the EAR/10^4^ PY Gy ≒ 2.3 observed after the Chernobyl accident. This suggests an underestimation of the thyroid dose by UNSCEAR of approximately 1/50~1/100 compared with the thyroid dose for Chernobyl. The increased childhood thyroid cancer in Fukushima was found to arise from radioactive iodine exposure, which was comparable to that in Chernobyl.

## 1. Introduction

After the radioactive fallout at the Fukushima Daiichi nuclear power plant (NPP) on 11 March 2011, the Fukushima prefecture initiated thyroid ultrasound examination (TUE) for all 367,649 residents aged ≤18 years at the time of the accident. The Fukushima Health Management Survey (FHMS) conducted by the Fukushima Medical University (FMU) reported 186 confirmed or suspected cancer cases in the first (fiscal year FY2011–2013) and second TUE (FY2014–2015) [1,2]. The FHMS further reported 316 confirmed or suspected cancer cases from the first to fifth TUE and the TUE every five years after the age of 20 years. The total number of thyroid cancer cases was 358 (303: confirmed by surgery) if confirmed thyroid cancer cases in the cancer registry outside FHMS were included (as of 20 July 2023).

The FHMS committee found that the incidence of thyroid cancer cases in the first and second TUEs was dozens of times higher than that estimated from the Japanese cancer statistics of an annual incidence of approximately 3 cases per 1 million for people younger than 20 years old [3,4,5]. The postoperative pathological diagnoses among 180 subjects who underwent operation for thyroid cancer in the FMU hospital by the end of 2018 revealed 175 cases of papillary thyroid carcinoma (PTC, 97%), 2 cases of follicular thyroid carcinomas, 2 cases of other thyroid carcinomas, and 1 case of poorly differentiated thyroid carcinoma [6] (pp. 42–43).

Thyroid dose after the Chernobyl accident was based on 350,000 direct thyroid measurements within several weeks after the accident [7] (B35). The United Nations Scientific Committee on the Effects of Atomic Radiation (UNSCEAR) 2020/2021 updated the thyroid dose estimates for 1-year-old infants to be 3.5–30 mGy in evacuation areas and 1.2–20 mGy in the rest of Fukushima prefecture [8], from the estimates of 33–83 mGy in the UNSCEAR 2013 report [9]. The UNSCEAR further concluded that a large excess of thyroid cancer diagnosed in FHMS would not be expected at the thyroid dose estimated by the UNSCEAR [8] (226(a)). It should be noted that thyroid dose in UNSCEAR 2020/2021 was not constructed from direct thyroid dose measurements but was estimated based on simulated ‘external + inhalation’ dose and constant ingestion dose.

The UNSCEAR declared that the large increase of thyroid cancer was not the result of radiation exposure, but was the result of ultrasensitive screening and consequential overdiagnosis, many of which may never result in clinical symptoms [8] (268 (q,r)). The definition of overdiagnosis by UNSCEAR was identical to the definition by Welch et al., i.e., any diagnosis that would not go on to cause symptoms or death [10]. However, no evidence was given except the underestimated thyroid dose by UNSCEAR.

Despite the high incidence of thyroid cancer after the nuclear disaster, the FMU has denied the association between radiation exposure and thyroid cancer in Fukushima. In the Special Issue on Fukushima in the Journal of Epidemiology in 2022, Shimura et al. of FMU suggested that the high prevalence of thyroid cancer diagnosed was not caused by radiation exposure, but rather by the highly sensitive detection method based on the UNSCEAR 2020/2021 [11]. Yasumura et al. said, “As quantifying overdiagnosis is challenging, the magnitude of overdiagnosis cannot be evaluated” [12]. Both the UNSCEAR and FMU ascribed the dozens-fold excess thyroid cancer in Fukushima to ultrasound screening leading to overdiagnosis, despite they admitted that the magnitude of overdiagnosis cannot be evaluated. Such an evaluation is likely outside the realm of science. It should be noted that fine-needle aspiration cytology (FNAC) was performed only for nodules larger than 5.1 mm in the FHMS, so the unnecessary FNAC and overdiagnoses should decrease with the use of ultrasensitive equipment.

The World Health Organization estimated the thyroid equivalent dose for 1-year-old infants to be 31–122 mSv in Fukushima prefecture in 2013 [13] (p. 43). However, the system for the prediction of environmental emergency dose information (SPEEDI) showed five areas of thyroid equivalent dose in the range of 100–10,000 mSv for 1-year-old infants and indicated that thyroid dose had reached 100 mSv even outside of the evacuation area [14] (p. 154). The National Institute of Radiological Sciences estimated thyroid equivalent doses from the data of Uno et al., 119–432 mSv among mothers and 330–1190 mSv in their infants living 45–220 km south or southwest of NPP [15,16]. Tsuda et al. claimed that owing to the large gap among the estimates, ranging from less than 1 mSv to more than 1000 mSv, alternative measurements are needed [17].

The UNSCEAR summarized the research on the relationship between thyroid cancer and estimated exposure levels. For the first TUE, two papers by Tsuda et al. observed radiation effects on thyroid cancer incidence [18,19]. Three research groups found associations between radiation dose and thyroid cancer incidence in the first and/or second TUE [20,21,22]. The FMU group found no statistically significant increase in thyroid cancer incidence with the exposure levels in the first [23,24,25] and second TUE [26,27]. However, the data in [23] showed a strong dependence on exposure levels if the annual incidence rate was compared by adjusting intervals from exposure to the examination of 0.8–2.2 years in the 1st TUE as in the second TUE [28]. The FMU could not find a statistically significant dose dependence because they adopted area divisions which did not reflect the exposure levels, as was evident from their maps of areas [24,25,26,27].

In our preceding studies, the thyroid cancer incidence of three individual external dose groups in the second TUE demonstrated a linear response to individual dose in the 0.5–2.5 mSv external dose range [29,30]. In this study, we quantitatively evaluated the dose–response relationship between the incidence rate of thyroid cancer (IR) per person-years (PY), IR/10^4^ PY of areas, and thyroid dose estimated in the UNSCEAR 2020/2021 report. By comparing the observed linear coefficients, i.e., the excess absolute risks per gray (EAR/10^4^ PY Gy) in Fukushima with those observed in Chernobyl, we obtain the calibration coefficient between the thyroid dose units of estimated dose in Fukushima and measured dose on thyroid soon after the Chernobyl accident. It can be understood why a dozens-fold increase of thyroid cancer incidence was observed in Fukushima despite the low thyroid dose estimated by UNSCEAR.

## 2. Materials and Methods

### 2.1. Subjects and Thyroid Ultrasound Examination

From the TUE for all 367,649 residents aged ≤18 years at the time of the accident, 115 and 71 confirmed or suspected cancer cases were detected among 300,473 and 270,511 examinees in the first and second TUE, respectively [1,2]. Among the 248 positive cases identified via FNAC (fine-needle aspiration cytology) in the first to fifth TUEs and TUE every 5 years, 247 cases (99.6%) were confirmed to be malignant by surgery and 1 was a benign tumor. In this study, the positive cases detected via FNAC were defined as cancer cases.

Each TUE consists of the primary examination and confirmatory examination. The ultrasound results in the primary examination are divided into three grades: A ((A1) without nodules/cysts and (A2) nodules ≤ 5.0 mm or cysts ≤ 20.0 mm), B (nodules ≥ 5.1 mm or cysts ≥ 20.1 mm), and C (immediate need for confirmatory examination). Grade A was scheduled for the next screening after 2 years; grades B and C underwent confirmatory examination, where advanced ultrasonography, blood, and urine tests were conducted. Positive cases in the confirmatory examination underwent FNAC or medical follow-up. When cancer cells were detected via FNAC, the patients were followed-up and underwent surgery at the appropriate time, where malignancy was finally confirmed [1,2].

The TUE dataset used in this study was deidentified and publicly available, so no ethical review was required.

### 2.2. Division of Fukushima Prefecture into Four Areas and Area Dose Estimation

**Figure 1:**
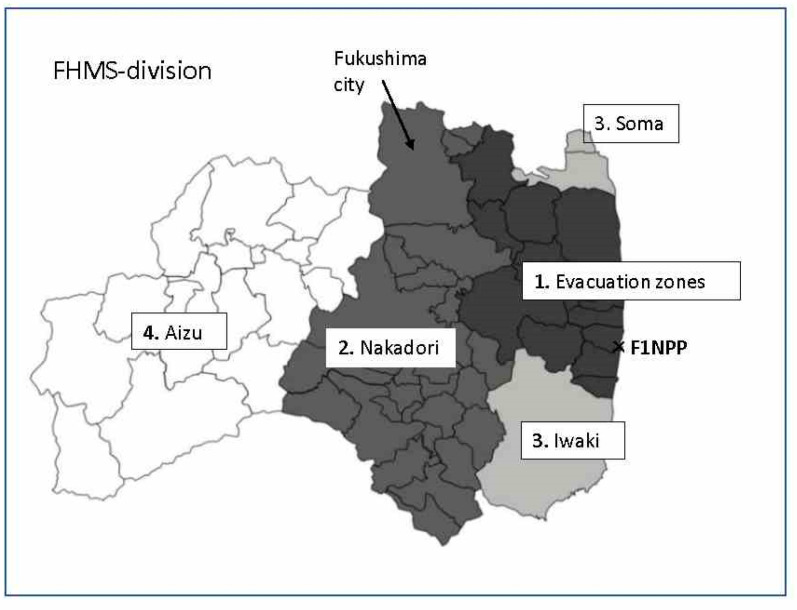
Map of 4 areas in the Fukushima health management survey (FHMS)-division of Fukushima prefecture.

Thyroid cancer incidence rates were estimated based on the division of the Fukushima prefecture in the FHMS reports (FHMS-division): (1) evacuation zone (highest dose), (2) Nakadori, (3) Iwaki and Soma, and (4) Aizu (the lowest dose) (Figure 1). The four areas, which are separated by two mountain ranges, roughly reflect the extent of radioactive contamination, e.g., the air dose rate and accumulation of ^134^Cs and ^137^Cs on the ground surface [31] (Attachment 1, 2). The four areas are similar other than their topography and radiation contamination, and no ecological fallacy was expected to affect the results.

The dose–response relationship was analyzed for the thyroid dose of 10-year-old children estimated in the UNSCEAR 2020/2021 report [8] (Attachment A-14, Table A-14.2). The thyroid dose in the report was not constructed from direct thyroid dose measurements but from simulated ‘external + inhalation’ dose and the ingestion dose from food and drinking water. The latter was assumed to be common in all municipalities in the Fukushima prefecture. The dose in each area was estimated using the average dose of each municipality weighted by the number of primary examinees in the first TUE [1,32]. In cases where multiple dose estimations were reported for a municipality in the evacuation zone, the highest value was employed [8] (A-18).

### 2.3. Thyroid Cancer Incidence Rates

An increasing trend in thyroid cancer incidence was recorded in the Belarusian cancer registry in the first year after the accident [33]. Howard reported that the minimum latency period for thyroid cancer in children younger than 20 years of age is 1.0 year [34]. Thyroid ultrasound examination was started in October 2011, and nodules ≥ 5.1 mm were examined for possible malignancy. The detected thyroid cancer nodules might have been smaller than those in clinical thyroid cancer soon after the Chernobyl accident. Thyroid cancer related to radiation exposure could be detected in the first TUE.

The FHMS committee evaluated the detected thyroid cancer cases in the first and second TUEs as dozens of times higher than that estimated from the Japanese cancer statistics for 2001–2010 [3,4,5,35]. In this study, we use thyroid cancer cases/10^4^ people: the prevalence proportion and incidence proportion defined in formal epidemiologic textbooks (e.g., Rothman [36]), multiplied by 10^4^ people for easy comparison with studies of the dose–response relationships after the Chernobyl accident. The observed thyroid cancer prevalence P1 (cancer cases/10^4^ examinees) in the first TUE was considered to be the sum of the prevalence just before the nuclear accident (P0) and the incidence proportion (IP1) from exposure to the first TUE, i.e., P1 = P0 + IP1. We approximated IP1 by the observed prevalence of P1, considering P0 was estimated to be approximately 1/60 (1.6%) of the observed P1 [3,4,5].

The mean interval years for each area after the accident to the first TUE (INT1) was estimated from the weighted average of the interval of each municipality (int1) by the number of primary examinees, where int1 was derived from the schedule of the FHMS [21]. The incidence rate per 10^4^ PY (person-years) in the first round, IR1, was estimated as IR1 = IP1/INT1. For the second TUE, the incidence proportion per 10^4^ examinees and incidence rate per 10^4^ PY were obtained from the data for those who underwent both primary examinations in the first and second TUEs [28].

### 2.4. Statistical Analysis

The dose–response relationship of the thyroid cancer incidence rate (IR) was analyzed using the linear excess absolute risk model in the form IR = r0 + r1 × dose, where r0 is the natural incidence rate from the cancer registry, r1 × dose is the excess absolute risk, and r1 is the excess absolute risk per Gray (EAR/Gy). The dose–response relationship of the IR was analyzed using regression analysis in Microsoft Excel 2019 MSO (2112). The confidence interval (95% CI) of the odds ratio of IR, compared with that in the least-contaminated area 4 as reference, which was estimated according to the formula by Rothman [36].

The lack of adjustment for age and sex, owing to the unavailability of the data in the FHMS, only had a slight effect on our results because the average age and sex (female %) were similar in the 4 areas.

### 2.5. Calibration between Thyroid Dose Units in Fukushima and Chernobyl

The estimated thyroid dose in UNSCEAR 2020/2021 for 10-year-old children (unit Gy^UN2021^) and the thyroid dose (unit Gy) based on 350,000 direct thyroid measurements within several weeks after the Chernobyl accident need to be calibrated [7] (B35). We estimated the calibration coefficient *k* (1 Gy^UN2021^ = *k* × 1 Gy) by comparing EAR/Gy: the linear dose coefficient of thyroid cancer incidence rates in Fukushima and Chernobyl after the nuclear accidents.

## 3. Results and Discussions

### 3.1. Thyroid Cancer Incidence Rate and Thyroid Dose in Different Areas

The thyroid dose in the four areas estimated for 10-year-old children from the UNSCEAR 2020/2021 report, thyroid cancer prevalence per 10^4^ examinees, annual incidence rate per 10^4^ PY, odds ratio (OR) of the incidence rate compared with the least contaminated area 4 as reference and its 95% CI for the first and second TUE are listed in Table 1.

### 3.2. Response of Thyroid Cancer Incidence Rate to UNSCEAR 2020/2021 Thyroid Dose

**Figure 2:**
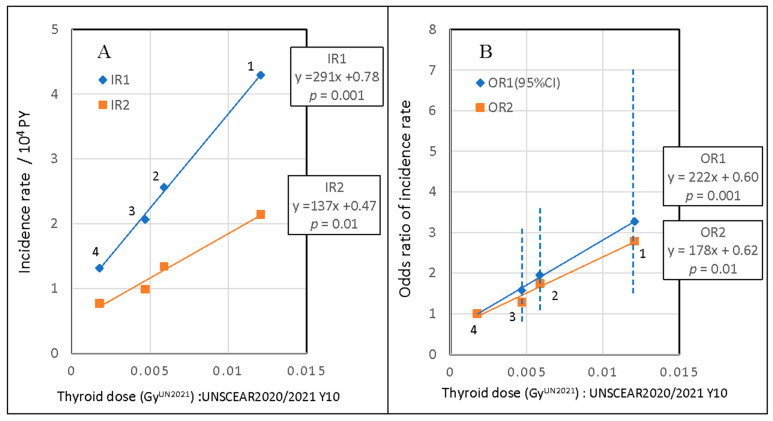
(**A**) Response of thyroid cancer incidence rates IR1 and IR2, and (**B**) odds ratio of the incidence rates, OR1 (95% CI) and OR2, to thyroid dose estimated for 10-year-old children in UNSCEAR 2020/2021 for four areas (1–4) in the FHMS (Fukushima health management survey)-division. Regression lines, formulae, and *p*-values for linear dose coefficients are provided.

The response of the incidence rate IR per 10^4^ PY to the thyroid dose estimated in the UNSCEAR 2020/2021 for 10-year-old children in the first and second TUEs is plotted in Figure 2A. The incidence rates IR1 and IR2 in the first and second TUEs showed a linear response to thyroid dose estimated in UNSCEAR 2020/2021 in the 0.002–0.012 Gy range. The odds ratios OR1 (95% CI) and OR2 in the first and second TUEs are plotted in Figure 2B. We approximated the incidence proportion IP1 after the accident by the prevalence P1 in the first TUE because P0 just before the accident was estimated to be approximately 1/60 of the observed P1 [3,4,5]. The linear dose–response of IR1 observed with *p* = 0.001 confirmed that the approximation was reasonable.

The observed linear relationship between the childhood thyroid cancer incidence rate in the study areas and thyroid dose showed that the increased thyroid cancer incidence detected in the first and second TUEs were associated with radiation exposure from the nuclear accident. In the histopathological analysis of patients with PTC who underwent surgical resection at FMU, Suzuki et al. found no statistically significant difference in tumor structure or invasiveness of the PTCs detected during 2012–2016, and suggested a common etiology of the PTCs detected in both TUEs [37]. This agrees with our conclusion of radiation exposure being associated with thyroid cancer in the first and second TUEs.

The incidence r0 at zero thyroid dose in the IR–dose plot was much higher than that expected from the data in cancer registries (Figure 2A). The UNSCEAR assumed thyroid dose via ingestion to be common to all municipalities in the Fukushima prefecture and revised it from 15 mGy in UNSCEAR 2013 to 0.95 mGy for 10-year-old children in UNSCEAR 2020/2021 without justifiable reason. The intercept r0 at zero dose decreased from positive to negative as ingestion dose increased. Hence, we added a base line dose (BLD) to ingestion dose so that the intercept r0 of IR agreed with the expected natural incidence rate from the cancer registry [30].

### 3.3. Comparison of Dose Dependence of EAR in Fukushima and Chernobyl after Nuclear Accidents

Jacob et al. found a linear increase in the EAR/10^4^ PY with thyroid dose in Chernobyl (Gy) during 1991–1995, where the expected cases were determined from the incidence in southern Ukraine of 0.042/10^4^ PY [38]. The EAR/10^4^ PY values of nine areas in Chernobyl were calculated from EAR/10^4^ PY Gy and the average thyroid dose in the area. The incidence rate is IR = r0 + EAR (r0 = 0.042/10^4^ PY for Chernobyl), where the IR and EAR are almost the same since the expected natural incidence rate r0 is negligibly small as compared with EAR in Chernobyl and in Fukushima. The thyroid dose and EAR/10^4^ PY after the Fukushima and Chernobyl accidents are compared in Table 2.

The results of the first and second TUEs showed the dozens-fold increase in childhood thyroid cancer compared with the cancer registry [3,4,5]. The ratio of the observed/expected cases of 50~60 in Fukushima prefecture in the 6 years after the accident was comparable to the 30~56 observed in high-dose areas in Ukraine and Belarus after the Chernobyl accident. This suggests a comparable exposure to radioactive iodine in Fukushima as in Chernobyl.

**Figure 3:**
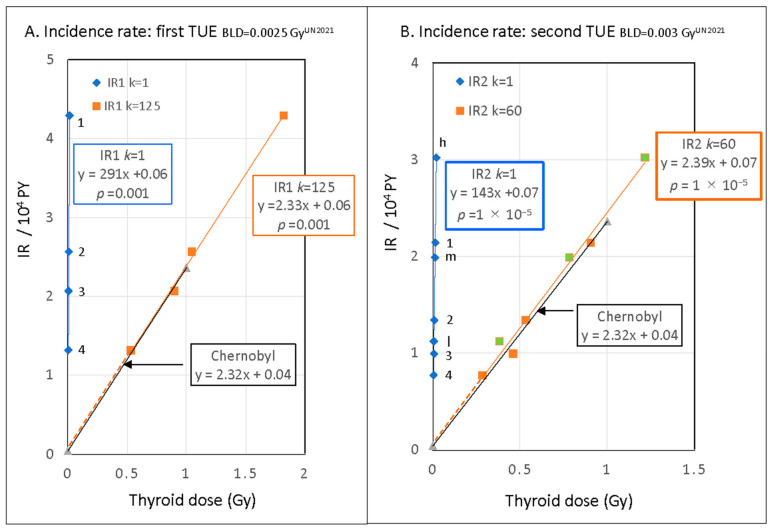
(**A**) Thyroid dose–response of IR/10^4^ PY of thyroid cancer of 4 areas in FHMS-division in the first TUE for BLD = 0.0025 Gy^UN2021^ (dose unit in Fukushima based on UNSCEAR 2020/2021). Blue rhombus: for 1 Gy^UN2021^ = 1 Gy (*k* = 1), and orange square: for 1 Gy^UN2021^ = 125 Gy (*k* = 125). The average linear response of IR (≒EAR) on thyroid dose after the Chernobyl accident (unit Gy) is shown by black line (y = 2.32x + 0.04). (**B**) Thyroid dose–response of IR/10^4^ PY in the second TUE for four areas (1–4) and three individual dose groups (h, m, and l) for BLD = 0.003 Gy^UN2021^. Blue rhombus: for 1 Gy^UN2021^ = 1 Gy (*k* = 1). Orange square for four areas, and green square of orange frame for three dose groups are for 1 Gy^UN2021^ = 60 Gy (*k* = 60). The average linear response of IR on thyroid dose after the Chernobyl accident is shown by black line (y = 2.32x + 0.04). Regression lines, formulae, and *p*-values for linear dose coefficients are given.

The dose–response of IR/10^4^ PY to thyroid dose estimated by the UNSCEAR 2020/2021 for 10-year-old children in Fukushima is compared with that in Chernobyl in Figure 3. For thyroid dose in Fukushima, we use adjusted thyroid dose with BLD value. The IR–dose plot in the first TUE for BLD = 0.0025 Gy^UN2021^ and *k* = 1, i.e., Gy^UN2021^ = Gy, is shown by the blue rhombus in Figure 3A. The nearly vertical blue regression line is y = 291x + 0.06, (*p* = 0.001), where x = dose and y = IR/10^4^ PY.

The dose response of IR after the Chernobyl accident was studied extensively [38,39,40,41], and the average EAR/10^4^ PY Gy value reported in the 25 years after the accident was 2.32 ± 0.17 (Table 3). The average linear response of IR (≒EAR) on thyroid dose (unit Gy) is shown by black line (y = 2.32x + 0.04) in Figure 3. The linear dose–response of the IR in Fukushima is quite different from that of Chernobyl. The observed EAR/10^4^ PY Gy^UN22021^ of 291 (95% CI: 248, 334) is approximately 125 (=291/2.32) times the average EAR/10^4^ PY Gy value of 2.32.

The IR–dose plot of the four areas and three individual dose groups, low (<1 mSv), middle (1–2 mSv), and high (≥2 mSv), in the second TUE [29,30] is shown in Figure 3B for BLD = 0.003 Gy^UN2021^ and *k* = 1 (blue rhombus). The observed EAR/10^4^ PY Gy^UN22021^ of 143 (95% CI: 122, 165) in the second TUE is approximately 60 (=143/2.32) times the average EAR/Gy value of 2.32 after the Chernobyl accident (Table 3). 

The high EAR/Gy^UN2021^ value in Fukushima compared with the EAR/Gy for Chernobyl cases suggests either that the carcinogenic sensitivity of children in Fukushima was approximately 60 times higher than that of children in Chernobyl or the thyroid dose estimated in the UNSCEAR 2020/2021 is a substantial underestimation of approximately 1/60. The IR–dose plot for *k* = 60 in the second TUE indicates an EAR/10^4^PY Gy^UN2021^ of 2.39 (95% CI 2.03, 2.75) (Figure 3B), in agreement with the average EAR/10^4^ PY Gy of 2.32 after the Chernobyl accident. Hence, *k* = 60 is considered to be a reasonable conversion coefficient.

The observed EAR/10^4^ PY Gy^UN2021^ of 291 and estimated calibration coefficient *k* = 125 (Figure 3A) in the first TUE are two times higher than those in the second TUE. This might be related to the high FNAC implementation rate of 26.5% in the first TUE compared with the 11.4% in the second TUE. If FNAC had been performed using similar criteria, the incidence rates and calibration coefficients of both TUEs would be more similar.

The thyroid cancer incidence rates in the first and second TUE showed a strong linear response to the thyroid dose estimated in the UNSCEAR 2020/2021. We further found that the thyroid dose was underestimated by approximately 1/50~1/100 in the UNSCEAR report compared with the thyroid dose determined based on direct thyroid measurements in Chernobyl (unit: Gy). In other words, the dozens-fold increase in childhood thyroid cancer after the Fukushima nuclear accident was found to arise from radioactive iodine exposure, which was comparable to that in Chernobyl.

Regarding the underestimation of thyroid dose by UNSCEAR 2020/2021, the estimated thyroid dose from inhalation of ^131^I using Atmospheric Transport, Dispersion, and Deposition Modelling (ATDM) is associated with considerable uncertainty, sometimes by orders of magnitude, at any given location, according to the response letter from UNSCEAR Chair Ms. Chen to S. Kurokawa [42]. As for the thyroid dose through ingestion, highly contaminated vegetables, e.g., spinach with 43,000 Bq/kg of ^131^I, were found in unpublished food data from the Fukushima prefecture [43]. The equivalent thyroid dose of a 5-year-old child from ingestion of 250 g of spinach every day until 20 March 2011, would exceed 100 mSv [17] (p. 13). This supports the high equivalent thyroid dose of 330–1190 mSv in infants via mother’s breast milk from the data of Uno et al., where six mothers out of seven were residents outside of the Fukushima prefecture [15,16]. The revised thyroid dose from ingestion by 1-year-old infants of 1.1 mGy in UNSCEAR 2020/2021 from 33 mGy (UNSCEAR2013) might be significantly underestimated [8,9]. Even if only the above two factors were considered, an underestimation of 1/50~1/100 by UNSCEAR seems probable. More details of the underestimation by UNSCEAR should be examined in the future.

### 3.4. Overdiagnosis Hypothesis without Evidence versus Possible Aggravation of Advanced-Stage PTC

Contrary to our result, both the FMU and UNSCEAR concluded that the high incidence of thyroid cancer diagnosed in the FHMS compared with the cancer registry was not the result of radiation exposure but rather the result of ultrasensitive screening leading to overdiagnoses [8,11,12,44]. However, confirmatory examination for B and C grades was performed only for nodules larger than 5.1 mm in the primary examination, so the unnecessary FNAC and resultant overdiagnoses should decrease with the use of ultrasensitive equipment.

The UNSCEAR estimated thyroid cancer risk in Fukushima using the small risk coefficient per unit dose observed in Chernobyl (mostly ERR/Gy) [41,45,46] multiplied by an underestimated thyroid dose in Fukushima assuming Gy^UN2021^ = Gy instead of the calibrated dose (1 Gy^UN2021^ = *k* Gy). They attributed the ‘observed high incidence of thyroid cancer minus the underestimated incidence of radiation-induced thyroid cancer’ to overdiagnosis, without any evidence. The real risk coefficient for Fukushima residents per unit Gy^UN2021^ was *k* = 50~100 times higher than the estimation by UNSCEAR. The risk coefficient EAR/Gy^UN2021^ or ERR/Gy^UN2021^ of Fukushima should be used, not that of Chernobyl, or thyroid dose calibrated to Gy in Chernobyl should be used. The ERR/Gy^UN2021^ of Fukushima was derived from the linear individual dose response of the relative risk [29,30].

The TUEs were conducted by the FHMS of FMU, and most surgeries were performed in the FMU hospital by Professor Suzuki. He reported 175 cases of PTC (97%) among the 180 cases undergoing operation at FMU; 72% showed lymph node metastasis and 1.7% showed lung metastasis [6] (pp. 42–43). He denied overdiagnosis in reporting that the operated cases did not include super low-risk cases, for which active surveillance is usually recommended. The pathological evidence was similar to that of thyroid cancer after the Chernobyl accident, i.e., short latency and aggressive characteristics with a high rate of lymph node metastasis, venous invasion, and elevated incidence of distant metastasis [47,48]. A journalist, Shiraishi, who attended Suzuki’s oral presentation at the 64th Annual Meeting of the Japan Thyroid Association in 2021 [49] reported as follows [50]. “Professor Suzuki reported that among 180 cases that he operated until December 2018, 16 cases received 19 RI treatments total, and 3 cases were scheduled for RI treatment. In the 16 cases, 9 cases had lung metastasis or suspected lung metastasis, one had suspected bone metastasis. Nine cases of lung metastasis are significant increase since only three cases were reported at the time of the first operation, and 10 distant metastases or suspected in 10 years (2~3 cases per million person-years) is surprisingly high. In addition, there were five other cases of metastatic and invasive lymph node involvement (N1-EX) and one case of lymph node metastasis in the lateral region. The concern from these cases does not appear to be ‘overdiagnosis’ but rather ‘severe aggravation’. The abstract was not open and recording and photography were prohibited in the annual meeting”. The publication of the paper from FMU is strongly urged because post-operative cases are essential to discuss the overdiagnosis hypothesis and the observed severe aggravations of advanced-stage thyroid cancer cases in young post-3.11 patients.

The FMU published “Merit and demerit of TUE” and requires parents of eligible people to understand the merits and demerits of TUE before making a decision to undergo TUE [51]. The possible demerits include the possibility of diagnosing and treating harmless thyroid cancer that may remain unnoticed for the rest of examinee’s life. It is explained further that it is not scientifically clear whether early detection of thyroid cancer by ultrasonography can reduce the cancer mortality rate. The public opinion that TUE should be stopped is increasing, influenced by the overdiagnosis hypothesis presented and spread by the UNSCEAR and FMU. It was reported that the incidence of thyroid cancer increased 3% annually in the United States during 1974–2013, with increases in the incidence rate and thyroid cancer mortality rate for advanced-stage PTC [52]. We should focus more on the possible aggravation of patients with advanced-stage PTC in the FHMS experiencing issues due to recurrence and metastasis.

The UNSCEAR 2000 explained that a confounding factor that did not correlate with dose could not be a bias in an epidemiological study of the effect of radiation dose on disease [53] (p. 301). This is consistent with “the properties of a confounding factor” by Rothman [36] (p. 141). If a strong positive correlation was found between the incidence rate of thyroid cancer and thyroid dose, any factors not associated with dose, including overdiagnosis, were unlikely to cause a high incidence of thyroid cancer. This applies exactly to the present case because strong positive correlations were found between incidence rate and thyroid dose in the first and second TUEs [17,18,19,20,21,22,29,30,32]. In addition to the observed dose–response relationship, much evidence was found of a radiation origin of PTC in Fukushima. First, among the 71 detected cancer cases in the second TUE, 33 were A1 “no nodules/cysts” in the first TUE, which suggested the high incidence in the second TUE due to the fast-growing nature of childhood PTC. Second, Demidchik et al. and Shibata et al. found that thyroid cancers were detected among children born before the Chernobyl accident, whereas no cases were detected by ultrasound screening among children born after the accident [47,54].

The observed strong association between IR and thyroid dose estimated by UNSCEAR thus indicated that the increased incidence of thyroid cancer among the exposed children in Fukushima was due to radioactive iodine exposure that was comparable to that of children in Chernobyl. Overdiagnosis can never be a substitute for radiation effect.

## 4. Conclusions

The origin of the dozens-fold increase in thyroid cancer via the thyroid ultrasound examination (TUE) of all residents aged ≤18 years at exposure to radiation from the Fukushima nuclear accident was studied. The observed linear response of the thyroid cancer incidence rates to thyroid dose estimated in the UNSCEAR 2020/2021 showed that the dominant origin of childhood and adolescent thyroid cancer incidence (97% PTC) detected in the FHMS in the 6 years following the nuclear accident was radiation exposure.

The EAR/10^4^ PY Gy^UN2021^ of 143 (95% CI: 122, 165) in the second TUE, where Gy^UN2021^ was the thyroid dose unit estimated by UNSCEAR 2020/2021, was approximately 50~100 times higher than the average EAR/10^4^ PY Gy ≒ 2.3 observed after the Chernobyl accident. This was due to the underestimation of the thyroid dose by UNSCEAR 2020/2021 by approximately 1/50~1/100 compared with the thyroid dose in Chernobyl (unit: Gy). The dozens-fold increase in childhood thyroid cancer incidence after the Fukushima nuclear accident was found to arise from radioactive iodine exposure that was comparable to that in Chernobyl.

The large increase in the thyroid cancer incidence rate in Fukushima, which increased proportionally to thyroid dose, was not the result of overdiagnosis, since overdiagnosis is not related to exposure dose. The screening effect and consequential overdiagnosis presented in the conclusion of the UNSCEAR 2020/2021 report were never proved. Young patients should not be made to wait because of overdiagnosis hypothesized without evidence.

## Figures and Tables

**Table 1:** Thyroid dose for 10-year-old children estimated in the United Nations Scientific Committee on the Effects of Atomic Radiation (UNSCEAR) 2020/2021 reports; first TUE (thyroid ultrasound examination), prevalence per 10^4^, interval years INT1 from the accident to the first TUE, incidence rate per 10^4^ PY, odds ratio of the incidence rate and 95% CI compared with reference area 4; second TUE, incidence proportion per 10^4^, incidence rate per 10^4^ PY, and odds ratio of incidence rate for areas in the FHMS (Fukushima health management survey)-division.

Area	Thyroid Dose	First TUE (FY2011–2013)						Second TUE (FY2014–2015) ^a^		
	2021, 10Y	Examinees	Cancer Cases	Prevalence per 10^4^	INT1	Incidence Rate per 10^4^ PY	Odds Ratio of IR1 (95% CI)	Incidence Proportion per 10^4^	Incidence Rate per 10^4^ PY	Odds Ratio of IR2 (95% CI)
	Gy			P1	Year	IR1	OR1	IP2	IR2	OR2
1. Evacuation zone	0.0121	41,810	14	3.35	0.78	4.29	3.27	5.31	2.14	2.78
							(1.5–7.1)			(0.9–8.3)
2. Nakadori	0.0059	169,155	65	3.84	1.50	2.56	1.95	2.77	1.34	1.74
							(1.1–3.6)			(0.6–4.9)
3. Iwaki Soma	0.0047	55,788	24	4.30	2.08	2.06	1.57	2.15	0.99	1.29
							(0.8–3.1)			(0.4–4.1)
4. Aizu	0.0018	33,720	12	3.56	2.71	1.31	1 (Ref.)	1.44	0.77	1 (Ref.)
Average /Sum	0.0061	300,473	115	3.83				2.62		

^a^ Data for those who underwent both primary tests in the first and second TUE [28].

**Table 2:** Average thyroid dose, observed/expected cases, and excess absolute risk EAR/10^4^ PY among children in Fukushima and affected areas after the Chernobyl accident.

Country and Area	Average Thyroid Dose (Gy)	Observed Cases /Expected Cases ^a^	EAR/10^4^ PY
Japan: Age at exposure 1~18, Examination period 2011~2015			
Fukushima prefecture	0.002–0.015	50~60 [3,5]	0.8~4
Chernobyl: Age at exposure 0~15, Examination period 1991~1995 (Table 1 [38])			
Ukraine: 30 km zone evacuees	0.92	30	1.20
Belarus: Gomel/Mogilev	0.73	56	2.34
Belarus: Gomel city	0.40	30	1.24
Other 6 areas in Ukraine, Belarus, and Russia: Bryansk	<0.2	<10	<0.32

^a^ Expected cases in Chernobyl were calculated on the basis of the incidence in southern Ukraine of 0.042 case per 10^4^ PY.

**Table 3:** (**A**) The EAR/10^4^ PY Gy^UN2021^ from the regression analyses of thyroid cancer incidence rates of areas (FHMS-division) and of individual dose groups in the first and second TUE (thyroid ultrasound examination) versus thyroid dose estimated by UNSCEAR 2020/2021 for 10-year-old children. The TUE was for residents aged ≤18 years at exposure. (**B**) Chernobyl cases.

**(A) Fukushima Prefecture** **Area Dose and Individual Dose**	**BLD/Gy^UN2021^**	**EAR (95% CI)** **/10^4^ PY Gy^UN2021^**	***p*-Value for** **EAR/Gy^UN2021^**
First TUE: 4 areas	0.0025	291 (248, 334)	0.001
Second TUE: 4 areas	0.003	137 (76, 198)	0.01
Second TUE: 3 individual dose groups	0.0045	137 (83, 191)	0.02
Second TUE: 4 areas + 3 individual dose groups	0.003	143 (122, 165)	0.00001
**(B) Chernobyl Countries**	**Age at Exposure/Years**	**EAR (95% CI)** **/10^4^ PY Gy**	**Reference**
Ukraine, Belarus, and Russia 1991–1995	0–15	2.3 (1.4, 3.8)	Jakob et al., 1998 [38]
Belarus and Russia 1991–1995	0–15	2.1 (1.0, 4.5)	Jakob et al., 1999 [39]
Ukraine and Belarus 1990–2001	0–18	2.66 (2.19, 3.13)	Jakob et al., 2006 [40]
Ukraine 2000–2007	<18	2.21 (0.04, 5.78)	Brenner et al., 2011 [41]
Chernobyl (average value)		2.32 ± 0.17	

## Data Availability

The data that support the findings of this study are available from the corresponding author upon request.

## Abbreviations

UNSCEAR	United Nations Scientific Committee on the Effects of Atomic Radiation
TUE	thyroid ultrasound examination
FHMS	Fukushima Health Management Survey
FMU	Fukushima Medical University
FNAC	fine-needle aspiration cytology
PY	person-years
EAR	excess absolute risk per 10^4^ PY
P1	thyroid cancer prevalence per 10^4^ person in the 1st TUE
P0	thyroid cancer prevalence per 10^4^ person just before the accident
IP1	incidence proportion per 10^4^ from exposure to the 1st TUE; P1 = P0 + IP1
IR1, IR2	incidence rate per 10^4^ PY in the first and second TUE
EAR/Gy	excess absolute risk per Gray
Gy^UN2021^	thyroid dose unit estimated in UNSCEAR 2020/2021 for 10-year-old children
BLD	base line dose
