# Correction: Kato et al. Area Dose–Response and Radiation Origin of Childhood Thyroid Cancer in Fukushima Based on Thyroid Dose in UNSCEAR 2020/2021: High ^131^I Exposure Comparable to Chernobyl. *Cancers* 2023, *15*, 4583

**DOI:** 10.3390/cancers15245802

**Published:** 2023-12-11

**Authors:** Toshiko Kato, Kosaku Yamada, Tadashi Hongyo

**Affiliations:** 1 Independent Researcher, Nara 630-8242, Japan; 2 Independent Researcher, Kyoto 611-0001, Japan; 3 Department of Radiation Biology, Health Sciences, Graduate School of Medicine, Osaka University, Osaka 565-0871, Japan; hongyo@sahs.med.osaka-u.ac.jp

The authors wish to revise two points in Sections 3.1 and 3.4. of this article [1]. First, in Table 1 column 2, line 3: 0.059 should be corrected to 0.0059.

Second, the original version of this article contained an incorrect and insufficient references in Section 3.4, paragraph 3. “Suzuki updated the data in 2021; out of 180 cases, 9 patients had lung metastasis or suspected lung metastasis, 1 had suspected bone metastasis (distant metastases total > 5%), 16 cases received RI treatment, and 3 cases were scheduled for RI treatment (>10%) [49,50]”. It should be corrected as follows:

A journalist, Shiraishi, who attended Suzuki’s oral presentation at the 64th Annual Meeting of the Japan Thyroid Association in 2021 [49] reported as follows [50]. “Professor Suzuki reported that among 180 cases that he operated until December 2018, 16 cases received 19 RI treatments total, and 3 cases were scheduled for RI treatment. In the 16 cases, 9 cases had lung metastasis or suspected lung metastasis, one had suspected bone metastasis. Nine cases of lung metastasis are significant increase since only three cases were reported at the time of the first operation, and 10 distant metastases or suspected in 10 years (2~3 cases per million person-years) is surprisingly high. In addition, there were five other cases of metastatic and invasive lymph node involvement (N1-EX) and one case of lymph node metastasis in the lateral region. The concern from these cases does not appear to be ‘overdiagnosis’ but rather ‘severe aggravation’. The abstract was not open and recording and photography were prohibited in the annual meeting”. The publication of the paper from FMU is strongly urged because post-operative cases are essential to discuss the overdiagnosis hypothesis and the observed severe aggravations of advanced-stage thyroid cancer cases in young post-3.11 patients.

The authors apologize for any inconvenience caused and state that the scientific conclusions are unaffected. This correction was approved by the Academic Editor. The original publication has also been updated.
